# Real-Time Magnetocardiography with Passive Miniaturized Coil Array in Earth Ambient Field

**DOI:** 10.3390/s23125567

**Published:** 2023-06-14

**Authors:** Keren Zhu, Asimina Kiourti

**Affiliations:** Department of Electrical and Computer Engineering, The Ohio State University, Columbus, OH 43210, USA; kiourti.1@osu.edu

**Keywords:** bioelectromagnetics, induction coils, magnetocardiography, medical sensing

## Abstract

We demonstrate a magnetocardiography (MCG) sensor that operates in non-shielded environments, in real-time, and without the need for an accompanying device to identify the cardiac cycles for averaging. We further validate the sensor’s performance on human subjects. Our approach integrates seven (7) coils, previously optimized for maximum sensitivity, into a coil array. Based on Faraday’s law, magnetic flux from the heart is translated into voltage across the coils. By leveraging digital signal processing (DSP), namely, bandpass filtering and averaging across coils, MCG can be retrieved in real-time. Our coil array can monitor real-time human MCG with clear QRS complexes in non-shielded environments. Intra- and inter-subject variability tests confirm repeatability and accuracy comparable to gold-standard electrocardiography (ECG), viz., a cardiac cycle detection accuracy of >99.13% and averaged R-R interval accuracy of <5.8 ms. Our results confirm the feasibility of real-time R-peak detection using the MCG sensor, as well as the ability to retrieve the full MCG spectrum as based upon the averaging of cycles identified via the MCG sensor itself. This work provides new insights into the development of accessible, miniaturized, safe, and low-cost MCG tools.

## 1. Introduction

Magnetocardiography (MCG) is a non-invasive and non-contact means of capturing the magnetic fields radiated by the human heart. Key clinical benefits of MCG compared to electrocardiography (ECG) entail: (1) the ability to provide three-dimensional mapping of the heart [1]; (2) high sensitivity toward tangential and vortex currents [2]; and (3) clear and reliable detection of cardiac activity through thick tissue (e.g., monitoring of fetal cardiac activity) given that magnetic fields propagate relatively undisturbed though body tissues (tissue permeability ≈ 0) [3]. In turn, several studies have demonstrated the superiority of MCG to monitor coronary artery diseases in patients without persistent ECG features, identify early repolarization patterns to prevent ventricular fibrillation, provide more accurate prognosis of ventricular tachycardia, test rejection reaction post heart transplantation, and detect various related fetal cardiac conditions [4,5,6,7,8,9].

The technologies that are available for capturing the extremely weak MCG signals (range of 50–100 pT) are summarized in [4]. In brief, clinical practice mainly uses superconducting quantum interference devices (SQUIDs) [10] that convert the cardiac magnetic flux into oscillating voltage via Josephson junctions in extremely low-noise environments. To achieve the desired performance, SQUIDs need to operate in a super conducting state (operating temperature of ~4 K to ~77 K) and in the presence of magnetic shielding. In turn, SQUIDs are bulky, non-portable, and expensive. Recent technology has introduced different types of atomic magnetometers (AMs), such as Optically Pumped Magnetometers (OPM) and Spin Exchange Relaxation-Free (SERF) magnetometers, to sense MCG signals. AMs achieve similar detection sensitivity as SQUIDs with much smaller size and without the need for cryogenics [11]. Nevertheless, AMs are active devices that require alkali atoms to be heated to a high temperature (~150 °C), raising safety concerns. In addition, most AMs can only operate in a near-zero magnetic field and their signal bandwidth is extremely limited [12]. Very few OPMs (such as the QuSpin Total Field Magnetometer, QTFM) can operate in earth ambient noise, but at the cost of degraded noise performance [13]. To date, no AM can record clear human MCG in the absence of shielding. The closest achievement entails the recording of heartbeat from cattle as based upon data recorded for a long period of time [13]. The research works reported in [14,15] overcome the key limitations of AM and they both achieve MCG detection in non-shielded environments. However, these approaches come with their own limitations: Ref. [14] does not demonstrate MCG detection in real-time while the device in [15] is not passive (i.e., it requires an additional external magnetic field to be applied).

To address these limitations in the state-of-the-art, we previously reported a fully passive, miniaturized, and light-weight sensor that can detect MCG signals in non-shielded environments, as validated upon phantoms [16,17]. The sensor operates on the principle of Faraday’s law where alternating magnetic flux from the heart interacts with induction coils placed upon the chest to induce voltages upon them. Through the theoretical optimization of the coil geometry and advanced digital signal processing (DSP), the final MCG signal can be retrieved in an earth ambient field [18]. This sensor is comparable in size, weight, and sensitivity to a typical AM sensor (i.e., ~centimeter, ~grams, and ~pT/√Hz, respectively); fully passive without the need of any heated alkali atoms nor any type of cryogenic; and capable of recording emulated signals that mimic the human MCG activity. However, three limitations of our prior work are that (a) the MCG sensor relies on extensive averaging over a long period of time (i.e., 15 min) that may be unsuitable for certain clinical applications, (b) the MCG sensor only operates in the presence of an ECG sensor (or equivalent) that is used to identify the cardiac cycles for averaging, and (c) the performance has only been validated in vitro using a function generator and an 8-shaped loop to emulate MCG activity.

In a major step forward, we herewith expand upon our previous work to demonstrate a low-cost sensing system that (a) monitors the full spectrum of MCG activity with: i. clear R-peaks in real-time and ii. fairly close P, T, QRS, and U waves over ~4.5 min of averaging, (b) enables stand-alone operation of the MCG sensor without the need for an accompanying ECG sensor, and (c) has been validated in vivo on human subjects. Here, “full spectrum” indicates the full-frequency spectrum information, and “clear R-peaks” are visually identified across the time-domain measurement. To verify that the signal we capture is indeed MCG and not chest wall vibration, we also conduct a proof-of-concept test with the MCG sensor placed at a distance away from the participant’s chest. Comparing our system to the state-of-the-art, the reported MCG sensor (1) exhibits very low operation cost (per use) and fabrication cost, unlike SQUIDs that cost USD ~5000 just to be turned on [19]; (2) does not require any accompanying structures to operate, unlike SQUIDs that require coolant to maintain an operating temperature of from ~4 K to ~77 K and unlike AMs that require heating/thermal isolation structures as well as shielding; (3) can detect the R-peaks of MCG in real-time serving as a self-sufficient device for subsequent averaging, unlike previous induction coils that require an accompanying ECG device to capture the R-peaks [14]; and (4) is fully passive without any lasers or strong magnetic fields needing to be applied, unlike AMs and [15].

The rest of the paper is organized as follows: Section 2 discusses the methods used to conduct our studies, including participant characteristics, recording system design, study protocol, and data processing. Section 3 reports and discusses results from our experimental validation and repeatability tests. Section 4 includes a discussion of our findings. The paper concludes in Section 5.

## 2. Materials and Methods

### 2.1. Participants

Eleven (11) healthy volunteers between 22 and 35 years (M = 26.4 years, SD = 4.2 years) of age were recruited for the experiment under approval from The Ohio State University Institutional Review Board (IRB) (#2019H0259) on 30 August 2019. The sample size of the recruited participants was selected to be comparable to other similar studies [20,21,22]. Demographic and physical characteristics data (age, gender, height, weight) were collected prior to conducting the experiments, with no personal identifying information.

Of the 11 enrolled participants, 4 identified as female (36%) and the other 7 identified as male (64%). The participant sample consists of females aged between 22 and 35 (standard deviation, SD = 5.6 years), height between 163 cm and 170 cm (SD = 2.9 cm), weight between 48.5 kg and 66 kg (SD = 7.2 kg), and Body Mass Index (BMI) between 18.3 kg/m^2^ and 22.8 kg/m^2^ (SD = 1.9 kg/m^2^). The male participants were aged between 23 and 30 (SD = 2.4 years), height between 165 cm and 192 cm (SD = 8.4 cm), weight between 55 kg and 82 kg (SD = 11.8 kg), and BMI between 19 kg/m^2^ and 26.1 kg/m^2^ (SD = 2.9 kg/m^2^).

### 2.2. Experiment System Design

**Figure 1:**
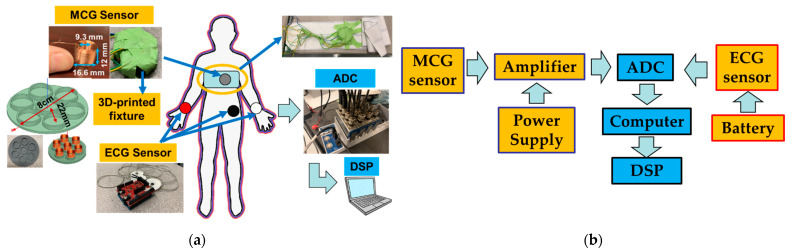
(**a**) Overview of the recording system; (**b**) Block diagram of the sensor and electronics connections.

The recording system employed in this study is shown in Figure 1a and consists of MCG and ECG sensors, an Analog to Digital Converter (ADC), and a laptop computer for storing and processing the data. The block diagram of how these components were connected is shown in Figure 1b and further described next. We remark that the ECG data were collected as the “gold-standard” electric equivalent of MCG with a goal to validate the performance of the MCG sensor. In real-world scenarios, cardiac activity can be ultimately monitored directly from the MCG sensor itself without the need for a concurrent ECG recording. This is a unique aspect vs. our previous work where an ECG sensor had to accompany the MCG sensor for identifying the cardiac cycles to be averaged [17].

**Figure 2:**
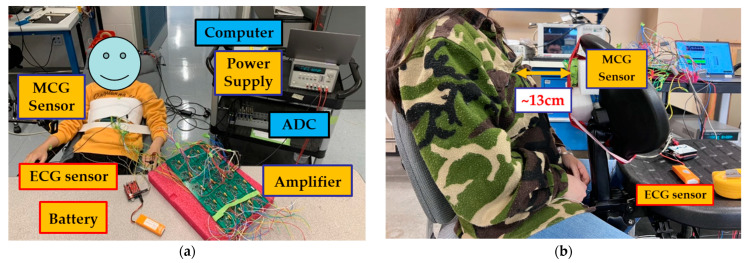
(**a**) In vivo experimental setup employed in this study; (**b**) MCG confirmation test setup.

The MCG sensor is based on the operating principle we previously reported in [16,18]. To achieve the target performance (i.e., real-time monitoring of the full MCG spectrum), we modified the MCG sensor design to entail 7 identical coils integrated into an array 8 cm in diameter. Each coil is of an optimal dimension in detecting the axial direction of the magnetic field, viz., Di (inner diameter)/D (outer diameter) = 0.56 and l (length or height of the coil)/D = 0.7182, as discussed in [16]. The exact coil parameters are selected as in [18] to match the high sensitivity performance, viz., Di = 9.3 mm, l = 12 mm, and D = 16.6 mm. The coils are based on an optimized tightly winded air-core coil design [16] with design parameters being represented by the coil geometry (i.e., outer/inner diameter, wire diameter, and coil height/length). Applying the tightly wound coil model, the number of turns is calculated to be ~1095. However, the exact number of coil turns are not provided to the coil manufacturer and the actual number of turns may slightly vary per the employed winding technique and fill factor. The total number of 7 coils is determined by first using a single-coil sensor to collect human MCG data as a function of time. With that, 7 cardiac cycles are identified as the minimum number of cycles needed to be averaged to see a clear MCG signal. This is due to the reduced level of uncorrelated noise, as discussed in [16,18]. With a goal to monitor the MCG data in real-time, instead of averaging over cardiac cycles, we herewith average over 7 different coil recordings that capture data at the same time. Since coils are small in size and placed in close proximity to each other, they often capture the same signal. Referring to Figure 1a (top left), 1 coil is placed in the center of the fixture and the remaining 6 are placed evenly surrounding it. The MCG sensor is covered with very thin and electromagnetically transparent green tape to keep the coils in place. It is then secured on to an elastic chest belt (Figure 2a, top left).

Compared to our previous design for MCG detection [16], our coil sensor is slightly larger with its geometry better fitting our previously calculated ideal ratio of dimensions. Specifically, the coil’s outer radius (D) changed from 15 mm (reported in [16]) to 16.6 mm, and the coil’s length (l) changed from 11 mm (reported in [16]) to 12 mm. The coil’s inner diameter (Di) and wire diameter (d) were kept the same as in [16]. The coils (seven in total) were arranged in a flower-like arrangement supported by a 3D-printed fixture with a total diameter of 8 cm. The fixture includes seven tightly fitted slots, with one for each of the coils. Specifically, six slots are centered along the edges of a hexagon and the seventh is placed in the center of the structure (Figure 1a, left). The distance between the center coil and each side coil is 22 mm. This design is selected to maximize the number of coils across a relatively small circular area as needed for both localized MCG detection and averaged real-time MCG detection. We note that the design of such fixture can be altered to better fit any specific application.

Seven (7) low noise amplifiers (gain = 1000) connect to each of the coils. They are all fabricated on a single circuit board and placed far away from the sensors to reduce the vibration and electronic noise. A detailed circuit design is discussed in [16], where the input network, voltage regulator, low noise instrumental amplifier (INA217), and reference pin offset correction (OPA2277) are the building blocks for the employed amplifier. All signal processing is performed digitally to reduce the additional noise. A power supply is constantly powering the amplifier board with ±10 V. The noise performance (noise spectrum density) of the sensing system (one coil sensor and one amplifier) is identical to that of the non-shieled environment reported in [18].

The overall cost is very low for our proposed sensing system. Each coil sensor costs less than USD 1 to manufacture and there is no additional cost for shielding/any types of coolant/any accompanying structures. The fixture holding the sensor is based on standard 3D-printing approaches, while the circuit board includes low-cost components entailing one INA 217 (USD ~6), one OPA 2277(USD ~5), voltage regulators (UA78L05ACDRG4 and MC79L05ACDG, USD ~1 total), and some additional resistors/capacitors/connectors. For one amplifier board, the total cost is USD ~38 and the price can be significantly reduced when ordered in bulk quantities.

The ECG signals are recorded using an off-the-shelf Arduino-based 3-lead ECG sensor (e-Health sensor [23]) powered by an Arduino battery. Both analog signals (ECG and MCG) are converted to digital ones using a 4-channel, 24-bits National Instrument ADC (NI9239) that samples at 5 kHz through a National Instruments LabVIEW interface. Since 8 channels are needed to concurrently record ECG and MCG data (1 for ECG and 7 for each of the MCG sensor coils), 2 identical ADCs (NI9239) are combined in a CompactDAQ chassis (NI cDAQ-9189) and perform simultaneous multi-channel data acquisition.

### 2.3. Study Protocol

The study experimental setup is shown in Figure 2. All tests are conducted at the ElectroScience Laboratory, The Ohio State University, Columbus, OH, USA. They take place in a regular Radio-Frequency (RF) laboratory environment with a decent level of electronic noise and no shielding.

The MCG chest belt was wrapped tightly around the participant’s chest with the sensor array located in front of the chest and sitting slightly to the left of the breastbone (grey circle in Figure 1a). This configuration minimizes noise due to body movement and maximizes the magnetic cardiac signal strength as captured via the MCG sensor. Both the amplifier board and the power supply are placed far away from the participant to reduce electronic noise (on a workbench in the front of the participant and a cart placed on the side, respectively, as shown in Figure 2a). Three foam-based disposable ECG silver/silver chloride (Ag/AgCl) solid gel electrode pads are adhered to the left-hand wrist (white circle in Figure 1a), right-hand wrist (red circle in Figure 1a), and the left-side lower edge rib cage (black circle in Figure 1a) to record the ECG. The neutral, positive, and negative leads from the ECG sensor are connected to the three pads, respectively. Here, an Arduino-based ECG is used as our “gold standard” for comparison between the MCG and ECG signals. We acknowledge that there are more advanced, clinical-grade ECG devices, yet this particular device is herewith used for its low cost and off-the-shelf availability. The ADC that simultaneously recorded the ECG and MCG signals, as well as the laptop computer that stored the data were placed on a cart next to the participant.

Two studies are performed back-to-back, each lasting ~4.5 min. First, the participant was instructed to lie back on a zero-gravity chair with no body movement. Here, we purposely eliminated body movement to reduce noise to the largest possible extent. We refer to this study as a “no movement study”. Next, the participant is instructed to sit in an upright position (without his/her back lying upon the chair) and tap upon a cell phone with RF features turned off. This setup is used to increase the noise due to motion and better mimic a real-world scenario. Despite operating the phone in “airplane mode”, our setup is by no means considered as “shielded”. This is because the RF signal from the phone lies outside the frequency range of the MCG signal. Even if it were present, this RF noise could easily be filtered out through DSP. The reason for operating the phone in “airplane mode” is to prevent any sort of distraction on behalf of the participants rather than shielding the experimental setup in any way. We refer to this study as a “minimal movement study”.

### 2.4. Data Processing

To derive the MCG signal, we employ two DSP methods: (1) bandpass filtering, and (2) averaging over different coils [18]. Compared to our previously proposed DSP [16], we eliminate the need for any accompanying trigger signal to identify the repeating cardiac cycles. In the past, to reduce noise, the MCG was recorded for a period of time together with a trigger signal (i.e., ECG). Using the R-peaks identified in the ECG, each cardiac cycle (1 s window containing one R-peak) was subsequently identified in the raw MCG signal. These identified cardiac cycles in the MCG were then averaged to produce the final clear MCG signal. Now, instead of ECG, averaging the MCG signal itself over seven (7) coils produces a clear R-peak that can be used as a self-trigger to identify repeating cardiac cycles. Here, the number 7 is identified as the minimum number of cycles needed to be averaged to see a clear MCG R-peak (per Section 2.2). Specifically, the raw MCG signals from each of the 7 coils, viz., *MCG_raw1…MCG_raw7*, first pass through a bandpass filter to reject the frequencies outside of the target frequency range (4–30 Hz), generating the signals *MCGf_1…MCGf_7*. The bandpass filter is implemented using the default MATLAB built-in “bandpass” function. It is designed as a minimal-order Finite Impulse Response (FIR) filter when the input signal (viz., *MCG_raw1…MCG_raw7*) is long enough, or a minimal-order Infinite Impulse Response (IIR) filter otherwise. In our case, all bandpass filters are FIR with 60 dB stop band attenuation and 0.1 pass band ripple. The delay of the filtered signal is compensated. The filtered signals (*MCGf_1…MCGf_7*) are then averaged based on the winding direction, as discussed next, to derive the final real-time MCG signal (*MCG_final_*).

Here, it is important to note that the winding direction of each of the coils needs to be considered in the averaging process. To determine the correct direction, a high-magnitude pre-recorded ECG signal is fed into a circular loop wire using a function generator and the converted magnetic signal is picked up by the proposed MCG sensing system. In this case, the raw signals captured by all 7 coils are large enough, such that the direction of winding for each coil can be determined without DSP. Specifically, we performed the calibration using a 4 cm radius single-turn circular loop wire fed by a function generator at 10 V pk-pk using a pre-recorded human ECG signal (the input impedance of the function generator being 50 Ω). All 7 coils are fixed onto the flower-shaped structure (per Section 2.2) to secure them in place and the whole structure is placed 15 mm away from the circular loop wire picking up the perpendicular magnetic field. With such a strong original signal, the signal that is being picked up can be clearly seen after averaging over repeating cycles (per [18]). This direction can be calibrated out during the coil averaging process by adding a negative sign to those signals with an opposite direction. With that, in our case, the *MCG_final_* is calculated as:

(1) $$MCG_{final}=\frac{\sum_{i=1}^{3}MCGf\text{\_}i-MCGf\text{\_}4+MCGf5-\sum_{n=6}^{7}MCGf\text{\_}n}{7}$$

To evaluate the performance of the MCG sensing system, we carry out two tests:

(1) *Validation test*. This test serves to validate (a) the feasibility of real-time MCG detection, and (b) the extraction of detailed MCG features through averaging over multiple cardiac cycles (or, equivalently, over a period). For this proof-of-concept validation step, we use data collected from the “no movement study” of a single participant (28 years old female; height 1.63 m, weight 48.5 kg, and BMI 18.3 kg/m^2^). Specifically:

(a) The real-time MCG detection is validated by confirming the presence of synchronized peaks in the ECG and *MCG_final_* signals. Here, synchronization refers to the ability of the major spikes of ECG and *MCG_final_* (known as R-peaks) to identify the same number of cardiac cycles across the same period. We remark that the exact alignment of the R-peaks between the ECG and *MCG_final_* signals is not anticipated in the time-domain as MCG is actually the derivative of ECG.
(b) Next, the MCG signals are averaged throughout the test duration, viz., across 4.5 min, to explore whether and which detailed features can be detected (such as P, T, and U waves). The process of averaging over repeating cycles has been described in [18] as a means of improving the MCG detection sensitivity. In the past, we used a concurrent ECG signal to serve as a trigger and identify the MCG cycles. Here, the ECG trigger is no longer required as the R-peaks can be detected in real-time using a stand-alone MCG sensor configuration. Hence, we can readily identify repeating cardiac cycles using the R-peak in the *MCG_final_* signal itself. We remark that averaging over the entire test duration (~4.5 min) is by no means limiting; shorter or longer averaging durations can be explored to derive the diverse features of the MCG signal.

(2) *Repeatability tests*. These tests entail intra-subject and inter-subject repeatability tests and serve to evaluate (a) the sensor’s detection accuracy vs. gold-standard ECG and (b) its tolerance to body movements. Specifically:

(a) Data recorded during the “no movement study” are used to analyze the sensor’s detection accuracy in terms of two different metrics, namely, QRS detection accuracy and average R-R interval accuracy. The former compares the number of visible QRS waves in the MCG signal (*MCG_QRS_*) as opposed to the number of visible QRS waves in the gold-standard ECG signal (*ECG_QRS_*) over the entire recording period and is defined as:

(2) $$Accuracy_{QRS}=\frac{MCG_{QRS}}{ECG_{QRS}}\times 100\%$$

To determine the average R-R interval accuracy, the average R-R interval is calculated for only those sections of time with 100% QRS detection accuracy (i.e., the same number of R-peaks detected in both the MCG and ECG). This is performed to eliminate the chance of missing cycles, which can lead to erroneous R-R interval calculations. Assuming that *n* number of R-peaks are detected within this timeframe and that *R_n_* represents the time when the corresponding R peak is identified, the average R-R interval is calculated as the sum of the times between all the two neighboring R-peaks divided by the number of R-R intervals.

(3) $$Average\;R\text{-}R\;interval=\frac{\sum_{i=1}^{n-1}R_{i+1}-R_{i}}{n-1}$$

*Average R-R intervals* are calculated for both MCG and ECG, viz., *MCG_R-R_* and *ECG_R-R_*, and the difference between the two is used as our second metric of accuracy for the MCG sensor:

(4) $$Accuracy_{R-R}=\left|MCG_{R-R}-ECG_{R-R}\right|$$

(b) The MCG sensor tolerance to movement is analyzed by comparing the data for the two study scenarios, i.e., “no movement study” and “minimal movement study”, throughout the test duration. The root mean square (RMS) of the isoelectric region and the minimum averaging time (or, equivalently, the minimum number of cardiac cycles) needed to identify the P and T waves are used to quantify the effect of the participants’ movement. Here, the isoelectric region, viz., the baseline region, is defined as the *MCG_final_* signal having excluded all visible QRS waves. Since motion artifacts occur within the spectral content of the original MCG signal [24,25], excluding the signal itself from the source signal (where signal, motion artifacts, and other sources of noise are combined), provides a signal that better represents the participants’ movement. Here, other sources of noise are assumed to be consistent across the two studies (i.e., “no movement study” and “minimal movement study”) as the data are collected one after another in almost the same time and environment. The QRS waves are excluded by deleting a 100 ms region across each detected R-peak, with 100 ms chosen as the widest QRS duration in healthy adults (per [26], QRS duration varies between 80 and 100 ms). Among standard measures of noise, viz., (1) root mean square (RMS) of isoelectric region; (2) ratio of R-peak to noise in the isoelectric region; (3) crest factor; (4) ratio between in-band and out-of-band power; and (5) power in the residual after filtering [27], the RMS of the isoelectric region is selected as the most accurate and direct means to represent the movement component of the noise. Additionally, (2) and (3) introduce the magnitude of the MCG signal into the equation, which complicate the process, while, for (4) and (5), the key component of the noise measured is the powerline noise (60 Hz), which lies outside the frequency of interest.

Finally, similar to the DSP procedure described in Section 2.5 (1) (b), cycle averaging is added to detect the detailed MCG features (i.e., P and T waves). Our hypothesis is that, with higher values of movement noise, the P and T waves will be harder to detect, leading to longer values of minimum time (or, equivalently, an increased number of cardiac cycles) needed for averaging. In turn, this minimum averaging time (or, equivalently, minimum number of averaging cardiac cycles) is herewith used as a parameter to quantify the noise. It is determined by gradually reducing the length of the *MCG_final_* vector used for cycle averaging until the P and T waves cannot be visually identified. The minimum length of *MCG_final_* that can retrieve visible P and T waves (*MCG_final__min*) is used to calculate the ‘*time_min_*’ and ‘*cycles_min_*’. Here, ‘*time_min_*’ is calculated as the minimal length (*MCG_final__min*) divided by the sampling rate of the ADC (*Fs*) and ‘cycles’ is defined as the number of R-peaks within the selected length.

(5) $$time_{min}=\frac{MCG_{final}\text{\_}min}{Fs}$$

### 2.5. MCG Verification

One possible concern that may arise with the wrap-around chest belt is the possibility of the resulting signal being attributed to chest vibration (similar to seismocardiography) as opposed to MCG. To verify that the signal is indeed MCG, we modified the previous setup so that the 7-coil sensor is placed a few centimeters away from the subject’s chest wall, i.e., none of the coils are touching the body. If the recorded signal is indeed MCG, it should be visible without any skin contact (though the increased distance from the chest may necessitate averaging over some extent of repeating cardiac cycles).

For this verification test, a 28-year-old female (height 1.63 m, weight 48.5 kg, and BMI 18.3 kg/m^2^) was recruited. Since the test only serves to confirm the MCG detection, only one participant is recruited as a proof of concept. The experimental setup is shown in Figure 2b, with the distance between the chest wall and the 7-coils array being ~13 cm. The materials surrounding this experimental setup (including the chair, table, etc.) are made of plastic, fabric, or foam, all of which have a relative permeability of close to 1. All the coils are pre-calibrated so that the corresponding winding is represented in the DSP, per Section 2.3, Equation (1). The coils are recording continuously, along with the 3-lead ECG system described in Section 2.3. In this case, ECG is used as our gating signal to identify the repeating cardiac cycles for averaging.

To keep it simple, DSP only utilizes three methods, namely, bandpass filtering, averaging over repeating cycles, and averaging over multiple coils, as discussed in [16]. Taking a step further, the time width of the QRS acquired during the MCG validation test is further evaluated to confirm whether the measured waveform is indeed MCG. Here, the QRS is measured manually in the final averaged signal. To compensate for the signal degradation due to the additional 13 cm distance, data are recorded for ~20 min, so that we have enough cycles for averaging.

## 3. Results

### 3.1. MCG Verification

**Figure 3:**
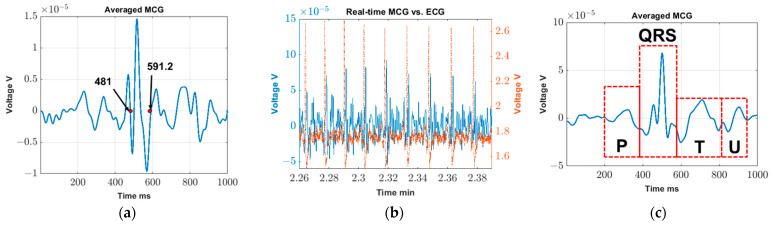
(**a**) MCG confirmation: cycle-averaged MCG over ~1 min of time recorded at 13 cm away from chest; (**b**) Validation test: Real-time MCG (blue solid line) vs. ECG (red dash line) in earth ambient noise; (**c**) Validation test: Cycle-averaged MCG over ~4.5 min.

Figure 3a shows the processed signal recorded 13 cm away from the chest. Expectedly, given the increased distance between the chest and the sensor, the MCG signal cannot be seen in real-time. Specifically, to generate the signal of Figure 3a, ~1 min out of the ~20 min recording is used (i.e., 75 cardiac cycles per coil). The QRS in the averaged MCG is manually measured to be 110.2 ms, which is within the normal range when compared to the results reported in [28,29]. We remark that, since this experiment only aims to confirm MCG detection, the setup is by no means optimized. Notably, the cardiac activity is detected by the sensor, confirming that the captured signal is indeed MCG.

### 3.2. Validation Test

#### 3.2.1. Real-Time MCG Detection

Figure 3b shows example MCG (blue solid line) and ECG (red dashed line) voltage data recordings in real time. The *x*-axis represents the time stamp. Though the study starts at 0 min and ends at 4.5 min, Figure 3b only shows data from 2.26 min to 2.39 min as an example. As seen, all R-peaks in the MCG can be clearly identified, and each peak is matched with a corresponding one in the gold-standard ECG. A similar correlation is found throughout the entire 4.5 min of the recording. This trend confirms our hypothesis that real-time MCG can indeed be captured with R-peaks that are clear in each cardiac cycle.

#### 3.2.2. Detailed MCG Feature Extraction

Figure 3c shows the averaged MCG over the entire study duration (i.e., ~4.5 min) which, in this particular recording, contains 313 cardiac cycles. We identify the cardiac cycles using the R-peaks of the MCG signal (see Figure 3b) and cut windows of 500 ms in duration in each side of the R-peak. In particular, the R-peaks are identified using the Matlab function “findpeak” with a defined minimal distance between the two peaks and defined minimal height of the peaks. In addition to the main QRS spikes, the P, T, and U waves can all be fairly closely identified in Figure 3c, confirming that, with minimal averaging (~4.5 min), detailed MCG features can be extracted.

### 3.3. Repeatability Test

#### 3.3.1. Detection Accuracy

The intra- and inter-subject QRS detection accuracy is calculated using Equation (2) and listed in Table 1 and Table 2, respectively. In the former case, a single participant (24 years old male; height 1.70 m, weight 55 kg, and BMI 19 kg/m^2^) repeats the “no movement study” seven different times. The QRS accuracy values (*Accuracy_QRS_*) are calculated using the entire data recording of ~4.5 min. The QRS/R-peak detection is again realized using the “findpeak” function in Matlab with a defined minimal distance between the two peaks and defined minimal heights of the peaks. As seen, clear MCG QRS waves are retrieved from all intra-subject trials and from 9 out of the 11 participants. In particular, subjects #7 and #9 are both female with thicker breast tissue and higher BMI (22.8 kg/m^2^ and 22.2 kg/m^2^, respectively), leading to increased distance between the signal source (heart) and the MCG sensor and, hence, lower signal quality. To put this in perspective, the two females that are included in this study have a BMI of 18.3 kg/m^2^ and 20.3 kg/m^2^, respectively. In this work, we exclude these two participants from subsequent data analysis, yet future advances in the hardware and signal processing will aim to improve the sensor performance. Referring to Table 1 and Table 2, the QRS detection accuracy among all intra- and inter-subject trials is >99.13%, with 5/7 trials (intra-subject) and 6/9 subjects (inter-subject) achieving 100% detection accuracy as compared to the gold-standard ECG.

The averaged R-R intervals (±SD) and the R-R accuracy are calculated using Equations (3) and (4) and are summarized in Table 3 and Table 4 for the intra- and inter-subject tests, respectively. Here, *Time_100%_* refers to the continuous time duration with a 100% QRS accuracy rate. Among all, the difference between the *MCG_R-R_* and *ECG_R-R_* is always <5.8 ms, with 6/7 trials (intra-subject) and 5/9 subjects (inter-subject) exhibiting 100% identical intervals. Comparing Table 3 and Table 4, the number of cases with *Accuracy_R-R_* = 0 are slightly higher for the intra-subject tests. On the other hand, compared within Table 3 and Table 4, the standard deviations for *MCG_R-R_* are slightly higher than those for *ECG_R-R_*. Nevertheless, for all intra- and inter-subject tests, the discrepancy between the averaged R-R intervals and the standard deviation calculated in MCG and ECG are negligible.

#### 3.3.2. Tolerance to Movement

Table 5 shows the RMS of the isoelectric region of the MCG in two scenarios, viz., no movement and minimal movement. As expected, higher RMS values are observed in all minimal movement studies due to the additional motion artifacts that are introduced into the recording. Here, data from the entire test duration (~4.5 min) are used to calculate the RMS value, and the 100 ms regions surrounding the R-peaks are deleted for those R-peaks that are detectable in Matlab. Since the MCG sensor has already proven to exhibit a QRS accuracy of >99.13% (see Table 1), only very few regions are left out. For each participant, the RMS values of the isoelectric region are different due to each test being conducted on different days with different environmental noise. Nevertheless, all values are in a similar level of signal strength (~10^−5^ V).

Table 6 shows the minimum time and minimum number of cycles needed to identify the detailed MCG features, specifically the fairly closed P, T waves, for each of the “no movement” and “minimal movement” studies. As would be expected, for all nine participants, a longer averaging time is needed to identify the P, T waves when retrieving MCG under minimal body movement. On average, the P, T waves can be identified by averaging over 23.1 s/30 cardiac cycles during the “no movement study” and 32.64 s/45 cardiac cycles for the “minimal movement study”. In all cases, the detailed MCG features can be extracted in less than 50 s/76 cardiac cycles. We remark that, since different participants have different heart rates, the number of cardiac cycles used for averaging is a better representation of the DSP process. For example, the participants with faster heart rates have an advantage in terms of the minimum time needed for average as, within the same period of time, more cardiac cycles are present.

**Figure 4:**
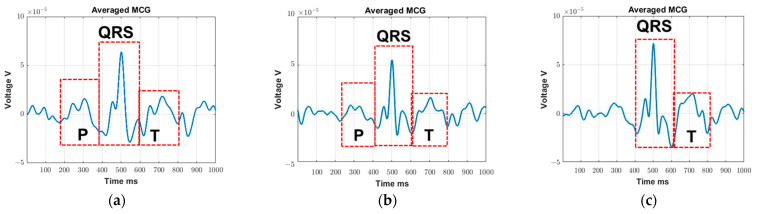
Example of processed averaged MCG using the minimum averaging time (from subject #1): (**a**) no movement and (**b**) minimal movement; (**c**) averaging over 13 cardiac cycles (same minimal cycles used in no movement study) for minimal movement study. Solid blue line represents the averaged MCG trace and the red dashed line represents the identified specific waves in the recorded MCG.

To better understand the impact of movement, Figure 4 shows the processed MCG plot for one of the participants: (a) using 11.40 s/13 cardiac cycles of averaging time for the “no movement study” (identified in Table 6 as the minimum time needed for averaging when no movement is present), (b) using 15.97 s/19 cardiac cycles of averaging time for the “minimal movement study” (identified in Table 6 as the minimum time needed for averaging when minimal movement is present), and (c) averaging over 13 cardiac cycles (same number of minimum cycles used for “no movement study”) for the “minimal movement study”. As seen, the P, T waves are quite visible in Figure 4a,b as most of the uncorrelated noise (i.e., motion artifacts) is eliminated with the increased averaging time in the second case. By contrast, the P wave is not visible in Figure 4c as the reduced number of averaging cycles is not sufficient to fully remove the motion artifacts. Here, the P wave is of a similar amplitude to that of the other waves in the isoelectric region. By contrast, the T wave is somewhat visible due to its higher amplitude. To sum up, regardless of the recording situation (e.g., “no movement” vs. “minimal movement”), the MCG signal can be retrieved with modifications in the recording time and the signal level for the same participant should be the same.

## 4. Discussion

The proposed real-time unshielded MCG detection system provides a promising solution for low-cost and continuous MCG monitoring outside the hospital setting. The results indicate the possibility of using such devices for (1) R-peak monitoring in real-time (e.g., to retrieve heart rate variability), (2) detailed MCG feature detection with ~4.5 min of averaging, and (3) MCG tracking with no and minimal movement.

**Figure 5:**
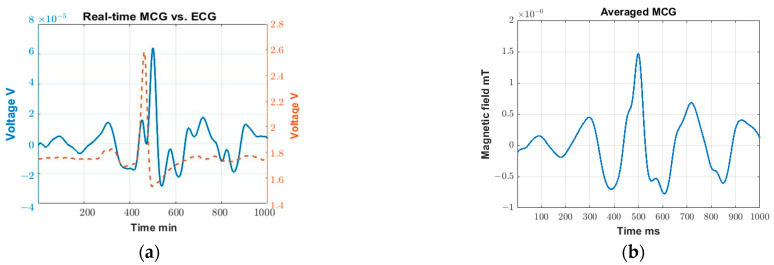
(**a**) Cycle-averaged MCG vs. ECG over ~4.5 min, and (**b**) Cycle-averaged MCG when converted to magnetic field unit (Tesla).

To confirm that detailed MCG features are indeed valid features and not artifacts, we superimposed the cycle-averaged ECG (red dashed line) on top of the cycle-averaged MCG (blue solid line) using the data obtained in the validation test (Figure 5a). Here, real-time R peaks (identified in *MCG_final_* per Section 2.3) are used as our trigger to identify repeating cardiac cycles for both ECG and MCG. As seen, only P-waves can be identified in the final cycle-averaged ECG signal as we are using a low-cost, off-the-shelf device. The location of this P-wave aligns with that of the MCG signal though, of course, and perfect alignment is not anticipated (note that the voltage induced is proportional to changes in the magnetic flux over time). Additional data regarding the time widths of QRS, PQ, and QT for all recruited subjects during the minimal movement study are included in Appendix A (Table A1) to further validate that the detected waveforms in all cases are indeed MCG. In the future, we expect that more extensive signal averaging and more advanced signal processing will further clear up the MCG signal, while clinical-grade ECG equipment will help demonstrate the correlation for other detailed features of the cardiac waveform (such as T and U waves).

The main goal of this study is to visualize the R-peaks of MCG in real-time so they can be subsequently used as a self-triggering signal. As such, we purposely select the 4–30 Hz frequency components of MCG as they contain most of the key features, particularly the R-peak. These detected R-peaks can then be used as a self-trigger for the MCG waveform filtered across a wider frequency range per application needs (e.g., 0.03–125 Hz [30] to identify ischemic ST elevation). Comparing Figure 3a with Figure 3c, the averaged MCG signal is obtained on the same subject with some distance (Figure 3a) and without any distance (Figure 3c) between the sensor and the chest wall. As seen, the R peaks can be detected in both cases, but with a reduced signal strength level when recording at a certain distance away. This is expected since the magnetic field strength produced by the heart is gradually degrading as the distance between the coil sensor and heart increases. In turn, detailed MCG features can barely be identified in Figure 3a.

**Figure A1:**
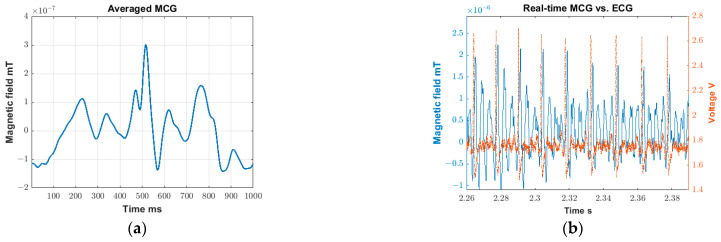
Converted magnetic field plot: (**a**) MCG confirmation: cycle-averaged MCG over ~1 min of time recorded at 13 cm away from chest, and (**b**) Validation test: Real-time MCG (blue solid line) vs. ECG (red dash line) in earth ambient noise.

Here, all MCG signals are represented in terms of voltage as a more straightforward representation, given that the cardiac magnetic flux is translated into voltage by the coil sensors. This voltage can be translated back to the magnetic flux (Tesla) by using a Helmholtz coil and providing a known uniform magnetic field in a shielded environment. The calibration can then be performed with the known magnetic field and the corresponding recorded voltage. When converting to magnetic field, the environmental and instrumental noise may complicate the process, making the converted results more prone to error. For instance, the uniform magnetic field produced by the Helmholtz coil can be altered by the additional noise present in the shielded environment. That is, the translation relies greatly on the effectiveness of the shielding. Since we are working with extremely low field strengths, even a small alteration in the background field can greatly impact the final results. That being said, Figure 5b shows an example plot with the signal converted from voltage to magnetic field using the data obtained in the validation test (Figure 3c), while the rest of the plots have been converted to a magnetic field and are included in Appendix A, Figure A1. Note that the converted signal strength (R-peak amplitude) in Figure 5b is in the range of 10^−9^ T (~1.5 × 10^−9^ T), which is bigger than the MCG amplitude typically expected from state-of-the-art sensors (~10^−10^T). This may be due to the distance between the heart and the recording sensor being smaller when compared to the typical recording setup using SQUIDs (>10 cm) [31]. In our case, the sensor is placed directly on top of the chest wall. Similar studies that utilize newer sensors recording at a shorter distance away from the heart have also shown similar results (i.e., R-peak values of ~10^−9^ T) [32,33]. As expected, the converted plot magnifies the lower frequency components and reduces the higher frequency components. Further, to perform such conversion (i.e., from voltage to magnetic field), Fast Fourier Transform (FFT) and Inverse Fast Fourier Transform (IFFT) need to be performed, which introduce additional error. This error becomes significant when dealing with weak signals. Along these lines, the manuscript shows the originally captured units (Volts) as the most representative means to represent the recorded MCG signal. As such, when voltage is used as the direct means of measurement, such conversion is not typically adapted in the literature [14,34,35,36].

We acknowledge that the current signal quality is far from ideal for clinical applications, especially when compared to already well-established MCG recording systems, such as SQUIDs or AMs [10,11,12,13]. As a comparison, current clinical grade SQUID systems can achieve a sensitivity of ~10–100 fT/√Hz around 10 Hz in shielded environments, whereas our current system can achieve ~30 pT/√Hz around the same frequency, yet in non-shielded environments. As such, some detailed MCG features, such as P and T waves are not as clear as those presented in SQUID-recorded MCG. In the future, bigger coils, ferrite cores, partial shielding, and even coolant approaches can be incorporated into the sensor to further improve its sensitivity. We also emphasize that the goal of this paper is not to retrieve the clearest possible MCG signal but rather to demonstrate the possibility of real-time R-peak detection as a subsequent self-trigger for clearing up the MCG signal per application needs. That is, the results presented in this work provide a starting point that opens doors for several sensor improvements and potential clinical opportunities in the future. We also note that, when retrieving detailed MCG features, the U wave is not always visible and the exact reason for this is still unknown and subject to research. We also remark that, when analyzing the effects of movement, as a proof-of-concept, the RMS of the isoelectric region is evaluated. This is by no means the optimal method for quantifying the noise introduced by the movement, as some of the MCG waveform information overlaps with the isoelectric region. Future work will focus on designing better parameters to quantify the impact of movement.

Comparing the intra- and inter-subject test results, the overall performance is better for the intra-subject tests, viz., the number of cases in which *Accuracy_R-R_* = 0 is higher as per Table 3 and Table 4. This can be explained by the fact that, as the participant performs multiple trials, they become more familiarized with the procedure, reducing motion artifacts in the recordings and leading to more accurate R-R interval values. Concurrently, for each subject and trials, *MCG_R-R_* tends to vary in values more than *ECG_R-R_*, viz., standard deviations for *MCG_R-R_* are slightly higher than *ECG_R-R_*. This may be due to the MCG signal, and particularly the MCG-derived R-peaks, being slightly distorted by noise. It is also worth noting that two female participants that have higher BMI (22.8 kg/m^2^ and 22.2 kg/m^2^, respectively) are excluded for all inter-subject studies due to low signal quality. Here, the higher BMI is not the sole reason for poor signal quality, but rather the key association lies in the distance between the heart and the sensor. For example, for male participants, even with higher BMI (subject #4 and subject #8, male, both having a BMI of 26.1 kg/m^2^), the MCG signal can still be retrieved. For this reason, the participants were divided not only based on BMI but also on gender. Considering this is a prototype recording system and a proof-of-concept experimental setup, more robust system hardware and better experimental setups will be explored in the future.

## 5. Conclusions

In this work, we presented a coil array that can detect the full frequency spectrum of a human MCG with visible R-peaks in real-time without any shielding. We further demonstrated the coil array’s ability to detect clear QRS detailed MCG features (P, T, and U waves) with ~4.5 min of averaging in non-shielded environments, without the need for any accompanying device to identify the cardiac cycles. The performance of the coil array was evaluated within and across subjects in terms of detection accuracy and tolerance to body movement. The in vivo experiments on human subjects confirmed that the detection accuracy for all intra- and inter-subject tests is >99.13%. The discrepancy in the averaged R-R intervals vs. gold-standard ECG is always <5.8 ms. When no movement is present, 23.1 s or 30 cardiac cycles are the minimal averaged time/cycles needed to identify the closed P, T waves. For minimal movement, these increased to 32.64 s and 45 cardiac cycles.

The reported sensor enables portable and low-cost MCG detection in real-time and non-shielded environments, showing promise for cardiac monitoring in the pre-clinical environment. In the future, leveraging e-textile technology, this device can be seamlessly integrated into garments enabling constant cardiac sensing.

## Figures and Tables

**Table 1:** Intra-subject QRS detection accuracy.

Trial Number (#)	*ECG_QRS_*	*MCG_QRS_*	*Accuracy_QRS_*
1	289	289	100%
2	230	228	99.13%
3	283	282	99.65%
4	273	273	100%
5	279	279	100%
6	279	279	100%
7	241	241	100%

**Table 2:** Inter-subject QRS detection accuracy.

Subject Number (#)	*ECG_QRS_*	*MCG_QRS_*	*Accuracy_QRS_*
1	315	314	99.68%
2	289	289	100%
3	307	307	100%
4	346	344	99.42%
5	248	248	100%
6	399	399	100%
8	344	343	99.71%
10	372	372	100%
11	411	411	100%

**Table 3:** Intra-subject averaged R-R interval and R-R accuracy.

Trial Number (#)	*ECG_R-R_* [ms]	*MCG_R-R_* [ms]	*Time_100%_* [s]	*Accuracy_R-R_* [ms]
1	1002.5 ± 47	1002.5 ± 68	289.7	0
2	1035.4 ± 70	1035.4 ± 92	190.5	0
3	1005.0 ± 63	1010.8 ± 94	180.9	5.8
4	962.7 ± 47	962.7 ± 143	262.9	0
5	1008.9 ± 69	1008.9 ± 114	281.5	0
6	927.9 ± 58	927.9 ± 58	258.9	0
7	1026.9 ± 59	1026.9 ± 64	247.5	0

**Table 4:** Inter-subject averaged R-R interval and R-R accuracy.

Subject Number (#)	*ECG_R-R_* [ms]	*MCG_R-R_* [ms]	*Time_100%_* [s]	*Accuracy_R-R_* [ms]
1	850.8 ± 38	850.8 ± 40	161.0	0
2	1002.5 ± 47	1002.5 ± 68	290.0	0
3	584.6 ± 41	588.4 ± 76	181.0	3.8
4	777.7 ± 45	777.4 ± 94	179.0	0.3
5	741.1 ± 42	741.1 ± 75	184.1	0
6	752.5 ± 43	752.5 ± 89	301.3	0
8	822.4 ± 23	825.2 ± 115	198.2	2.8
10	810.8 ± 57	810.8 ± 59	301.8	0
11	747.0 ± 46	748.8 ± 102	308.0	1.8

**Table 5:** RMS of isoelectric region: no movement vs. minimal movement.

Subject Number (#)	No Movement [μV]	Minimal Movement [μV]
1	16.59	16.87
2	17.44	22.18
3	29.88	37.70
4	18.93	22.58
5	20.81	22.36
6	21.96	22.99
8	17.73	19.52
10	29.94	36.57
11	29.94	40.10

**Table 6:** Minimal averaging time: no movement vs. minimal movement.

Subject Number (#)	No Movement		Minimal Movement	
	*Time_min_* [s]	*Cycles_min_*	*Time_min_* [s]	*Cycles_min_*
1	11.40	13	15.97	19
2	24.40	25	40.23	49
3	29.30	47	31.86	63
4	21.69	27	31.42	41
5	20.36	27	26.47	37
6	29.05	39	36.32	45
8	29.66	36	40.86	49
10	12.25	15	21.04	29
11	29.77	40	49.61	76
Average	23.10	30	32.64	45

## Data Availability

Not applicable.

## Appendix A

**Table A1:** Time widths of QRS, PQ, and QT in ~5 min averaged MCG for no movement study.

Subject Number (#)	QRS [ms]	PQ [ms]	QT [ms]
1	116.2	172.4	463
2	117.2	148.6	375.4
3	118.8	214	486
4	81.8	207.2	438.4
5	100.8	168.4	372.4
6	84.4	127.4	422
8	104	131.6	383.6
10	100.4	177.4	417.8
11	79	136.4	433
